# Preserving Smart Objects Privacy through Anonymous and Accountable Access Control for a M2M-Enabled Internet of Things

**DOI:** 10.3390/s150715611

**Published:** 2015-07-01

**Authors:** José L. Hernández-Ramos, Jorge Bernal Bernabe, M. Victoria Moreno, Antonio F. Skarmeta

**Affiliations:** Department of Information and Communications Engineering, Computer Science Faculty, University of Murcia, Murcia 30100, Spain; E-Mails: jorgebernal@um.es (J.B.B.); mvmoreno@um.es (M.V.M.); skarmeta@um.es (A.F.S.)

**Keywords:** privacy, access control, Internet of Things, anonymous credential systems, certificateless cryptography

## Abstract

As we get into the Internet of Things era, security and privacy concerns remain as the main obstacles in the development of innovative and valuable services to be exploited by society. Given the Machine-to-Machine (M2M) nature of these emerging scenarios, the application of current privacy-friendly technologies needs to be reconsidered and adapted to be deployed in such global ecosystem. This work proposes different privacy-preserving mechanisms through the application of anonymous credential systems and certificateless public key cryptography. The resulting alternatives are intended to enable an anonymous and accountable access control approach to be deployed on large-scale scenarios, such as Smart Cities. Furthermore, the proposed mechanisms have been deployed on constrained devices, in order to assess their suitability for a secure and privacy-preserving M2M-enabled Internet of Things.

## 1. Introduction

The so-called Internet of Things (IoT) [1] promises the realization of pervasive computing and ambient intelligence foundations, making IT an integral component of our daily lives [2]. In recent years, different technologies, such as IPv6 or Bluetooth, have been developed to enable a seamless integration of any physical device to the Internet infrastructure, enabling a continuous and transparent data communication among smart objects [3]. After the main technological challenges have been overcome, the increasing attention from academia and industry is encouraging the development of innovative scenarios based on this emerging paradigm.

In such digital revolution, the big challenge lies in the design of secure and privacy-preserving services, which will be deployed in everyday scenarios as an essential factor of Smart Cities [4]. Indeed, the *anytime*, *anything* and *anywhere* nature of the IoT raises serious security and privacy concerns, since highly sensitive information will be exchanged and managed in common IoT scenarios [5]. Such issues have been widely considered on current Internet scenarios, taking into account aspects, such as *Personally Identifiable Information* (PII) minimal disclosure [6], as a basic aspect of the *Privacy by Design* (PbD) notions [7]. However, the application of privacy-preserving mechanisms in IoT scenarios has to face significant challenges related to the need for managing billions of heterogeneous devices in open, distributed and dynamic environments, as well as the needs of citizens who will use these services. Indeed, the foundational principles of PbD are especially difficult to be satisfied in an interconnected global world. On the one hand, people demand richer experiences through more customized services being provided by any smart object. On the other hand, companies require highly sensitive information from users (e.g., location) in order to provide more satisfactory services. Therefore, IoT stakeholders' needs pose conflicting requirements that must be adequately regulated by legal concerns beyond the IT field. Therefore, traditional identity management and privacy-friendly solutions must be adapted, in order to stimulate the adoption of new and valuable services to be exploited by society [8].

Given the current lack of mature and suitable approaches for IoT environment, this work provides the design and development of different alternative mechanisms in order to address these challenges. Specifically, these methods have been designed on top of our *Distributed Capability-Based Access Control* (DCapBAC) [9] proposal, which was recently postulated as a feasible access control mechanism to support the life cycle of smart objects with tight resource constraints [10]. DCapBAC provides a flexible and efficient approach, in which constrained smart objects are enabled with authorization logic for an end-to-end access control solution. However, DCapBAC does not consider privacy aspects, since it is based on the use of traditional X.509 certificates, which are used to identify smart objects. In this direction, the main purpose of this work is to address the previous challengess by enhancing our DCapBAC approach with privacy features. For this purpose, we consider recent cryptographic schemes based on *Certificateless Public Key Cryptography* [11], and *Anonymous Credential Systems* (ACS) [12]. Specifically, we explore the application of *Identity-Based Encryption* (IBE) [13] and *Ciphertext-Policy Attribute-Based Encryption* (CP-ABE) [14] cryptographic schemes, as well as Idemix [15], as the most predominant example of ACS, in order to enable a privacy-preserving access control mechanism for the IoT. Furthermore, a common aspect of the proposed alternatives is that, while smart objects' privacy is preserved, our design enables the anonymity condition can be revoked in case of misuse or abuse. Moreover, unlike well-known mechanisms of the current Internet, *Trusted Third Parties* (TTP) are not required during smart objects interactions, fostering a highly distributed view as an integral part of a secure and privacy-preserving M2M-enabled IoT [16,17]. Currently, M2M foundations are considered as an essential aspect for a broad adoption of the IoT, enabling a direct communication between smart objects in an autonomous and natural way. The proposed mechanisms have been designed according our *Architectural Reference Model* (ARM) compliant security framework [18], which is intended to provide a holistic security and privacy-preserving approach for the IoT. Additionally, they have been implemented on constrained devices, and evaluated by considering performance and privacy aspects in order to assess their feasibility and suitability to be deployed on IoT scenarios.

The remainder of this paper is structured as follows. Section 2 describes some recent proposal addressing access control and privacy-preserving mechanisms, as well as the main background technologies that are used in our proposal. In Section 3, we briefly describe the required interactions between the components of our security framework for the IoT, in order to realize the different approaches. Section 4 details the three proposed alternatives for anonymous access control in the IoT. Section 5 provides a set of experimental results of the alternatives presented, which are compared and discussed in Section 6. Finally, in Section 7, we end up with some conclusions and an outlook of our future work in this area.

## 2. Related Work and Background

The realization of the IoT ecosystem imposes significant restrictions on security and privacy, since physical objects are being seamlessly integrated into the Internet infrastructure, through the use of different wireless communication technologies. In particular, IoT scenarios are intended to manage particularly sensitive data, and consequently, any information leakage could seriously damage the user privacy. This problem is aggravated in the IoT, since any smart object will be able to create new information and communicate it to any other entity. In the current Internet, identity management has been traditionally addressed by using well-known technologies, such as the *Security Assertion Markup Languaje* (SAML) [20], OpenID [21] or WS-Federation [22]. In these mechanisms, an *identity provider* (IdP) entity is usually in charge of creating on-demand access token, in order to enable a secure communication between two entities. This aspect hinders the adoption of security and privacy-preserving mechanisms for M2M scenarios, since the IdP is involved in all transactions between any two users. Therefore, such communications are linkable against this entity. In contrast to these technologies, another traditional approach is based on the usage of a *Public Key Infrastructure* (PKI) by employing X.509 certificates. These credentials are then used to realize typical authentication mechanisms (e.g., based on *Transport Layer Security* (TLS) [23]) in order to make communications secure among Internet nodes. An important advantage related to the use of X.509 certificates is that they allow an entity to prove the possession of a certain credential, without the need to involve the IdP in each communication. Therefore, it cannot link different transactions being made by a specific entity. However, interacting parties are unequivocally identified because of the use of certificates that are entirely disclosed to the other party, and consequently, unlinkability cannot be preserved.

Unlike previous alternatives, *Anonymous Credential systems* (ACS) [12] allow a selective disclosure of identity attributes to achieve a privacy-preserving identity management approach. Indeed, a user or entity can prove a specific set of properties associated to a subset of identity attributes, without disclosing the content of such attributes itself. Furthermore, while a *Trusted Third Party* (TTP) is responsible for generating the corresponding anonymous credentials, they enable M2M communications by making use of such credentials. A crucial aspect of privacy-preserving mechanisms is related to the design of mitigation strategies to avoid anonymity abuse, by considering *traceability* or *accountability* aspects. This property allows an entity to adopt counter-measures in case an attack or misuse of the anonymity is detected.

However, currently there is a lack related to the application of proper mechanisms for IoT scenarios, based on the privacy features that are provided by such technologies. In this work, we consider the application of ACS for the design and development of a privacy-aware access control mechanism for the IoT, by using anonymous authorization tokens. Such credentials are linked to specific subset identity attributes, which can be provisioned and proved in a privacy-preserving way. Additionally, we provide the design and evaluation of other alternative approaches based on recent certificateless cryptographic schemes, in order to provide a desired level of privacy to be leveraged in the development of M2M-enabled IoT services.

### 2.1. Related Work

The application of security and privacy-preserving mechanisms in this area is receiving an increasing interest from academia [24–27]. In particular, [28] presents the application of the *Usage Control* (UCON) model in IoT. They provide different examples to verify the expressiveness of the model by using fuzzy logic, although an evaluation of the proposed mechanism is not provided. The approach presented in [29] provides an authorization framework based on SAML assertions as a result of an authorization process based on the *eXtensible Access Control Markup Language* (XACML) [30]. These assertions are used to get access to an IoT device through the use of symmetric key cryptography. An Object Security Architecture (OSCAR) for the IoT is proposed by [31], as a scalable approach enabling an end-to-end secure access control mechanism based on public key cryptography to be deployed on constrained devices. While different evaluation results related to cryptographic operations, a detailed overview of the access control mechanism is not provided. The application of the capability-based access control model was proposed in [32], as a promising approach to be considered for emerging scenarios. Other proposals have considered the application of security mechanisms based on this model [33]. Nevertheless, they do not consider the implications related to the use on constrained devices in IoT scenarios. In particular, SAML is a well-known technology for the exchange of certified attributes enabling SSO and identity federation in inter-domain scenarios. Additionally, it provides authorization decision statements to transport authorization decisions instead identity attributes. Unlike the original DCapBAC approach in which access rights are associated to a public key (like in SPKI [34]), the format of these assertions are similar to the resulting anonymous tokens that are considered in this work. While the proposed alternatives can be used together these authorization assertions, the main focus of this work is the design of different mechanisms to prove the possession of an authorization credential in a privacy-preserving way, without the need to involve a third entity during the communication. Furthermore, the use of SAML presents several implications. On the one hand, SAML is based on the use of RSA signing and verification for public key operations [20]. In contrast, DCapBAC proposes the use of ECC for cryptographic operations, which enables to use smaller tokens due to the advantages of ECC in terms of key size. On the other hand, DCapBAC tokens are represented by using JSON as a lightweight alternative format to XML, which is employed by SAML. Moreover, DCapBAC defines a simple and extendable mechanism to transport conditions to be verified by target devices. Furthermore, access rights contained in DCapBAC tokens are mapped to CoAP methods to facilitate the verification process in the target device. DCapBAC is based on the main foundations of previous proposals, and it is used in this work as a basis for the design of a privacy-preserving access control approach for IoT smart objects.

The global interconnectivity envisioned by the IoT paradigm implies a huge amount of personal data to be disseminated and shared, with the aim to provide more advanced services to users. The set of privacy implications in IoT is considered by [35], proposing a set of trust-enhancing security functional components for the IoT resolution infrastructure under core notions of the IoT-A project [36], in order to provide basic security and privacy aspects in IoT communications. Moreover, [37] proposes the use of a two-factor authentication scheme based on public key cryptography. They discuss different practical solutions for realizing users' anonymity, and they demonstrate the complexity related to the design of privacy- preserving solutions based on such schemes. Additionally, [38] presents a distributed target-driven anonymous authentication protocol for IoT applications. This proposal is based on a multi-show credentials system, which is used by users to authenticate anonymously. An authentication and key agreement scheme is given by [39] for ad hoc WSNs. The proposed mechanism enables a remote user to securely negotiate a session key with a sensor node, ensuring mutual authentication and user anonymity by using masked identities. An aggregated-proof based hierarchical authentication scheme (APHA) for the Internet of Things is presented in [40]. This scheme is based on the use of homomorphism functions and Chebyshev chaotic maps for mutual authentication, as well as aggregated-proofs to achieve anonymous data transmission.

Regarding the application of the background technologies that are used in our proposed mechanisms, [41] considers the use of certificateless public key cryptography for IoT scenarios, proposing a lightweight scheme on the elliptic curve over the ring. Moreover, [42] considers the use of identity-based cryptography for secure communication between two smartphones that are paired with a Bluetooth NFC-based process. However, evaluation details are not provided. [43] highlights the growing interest on the attribute-based encryption mechanism due to its high potential to be applied in different areas. In this direction, [44] proposes a lightweight ABE scheme to be implemented on constrained devices, by considering the use of elliptic curve cryptography. An attribute-based signature scheme (ePass) is proposed in [45], which demonstrates user anonymity and attribute privacy. The application of CP-ABE to manage security and privacy aspects in heterogeneous medical wireless body area networks is proposed in [46]. They present two protocols for publishing data and sending commands to a sensor that guarantee confidentiality and fine-grained lattice-based access control (LBAC). Moreover, the application of anonymous credential systems in different scenarios is being investigated under several European projects, such as ABC4Trust [47]. However, they are not being considered in emerging scenarios of the Future Internet. In this work, these technologies are considered as alternative privacy-preserving identity management mechanisms, in order to design and evaluate an anonymous access control mechanism for IoT scenarios.

### 2.2. Preliminaries

Before the detailed explanation of our proposal, for the sake of readability, in this section we provide a brief description of some concepts related to the main technologies that make up our alternative mechanisms.

#### 2.2.1. DCapBAC

As already mentioned, the application of security and access control mechanisms in IoT must deal with strong requirements related to interoperability, flexibility and scalability. Recently, we proposed the *Distributed Capability-based Access Control* (DCapBAC) model [9], as a feasible access control approach to be deployed even on devices and networks with tight resource constraints. DCapBAC is based on the use of authorization tokens, containing access privileges that were previously granted to the holder, as well as a set of access conditions to be locally verified at the end device. Under the main foundations of *AuthoriZation-Based Access Control* (ZBAC) [48], SPKI *Certificate Theory* [34] and the *Policy Machine* from NIST [49], DCapBAC allows a distributed approach in which IoT devices are enabled with authorization logic by adapting the communication technologies and representation format. Specifically, it makes use of the *JavaScript Object Notation* (JSON) [50] as representation format for the capability token, which are attached on access requests by using the *Constrained Application Protocol* (CoAP) [51]. Furthermore, it defines an optimized *Elliptic Curve Cryptography* (ECC) library, in order to provide an access control mechanism for the IoT, while end-to-end security is preserved.

DCapBAC has been extended in our previous work [52] to enable a trust-aware access control mechanism, by considering trust values as a component to drive the authorization logic of smart objects. However, although DCapBAC provides a secure, flexible and efficient access control mechanism for IoT environments, it does not propose any privacy-preserving feature that enable smart objects to make use of capability tokens anonymously. In particular, the DCapBAC approach is based on linking access privileges to individuals, who are identified by their public key (which is usually contained in a X.509 certificate) [34]. Therefore, a subject is unequivocally identified when it tries to access a resource using such credential. In this direction, the main purpose of this work is to enrich the DCapBAC proposal with privacy-preserving features, by proposing and evaluating different alternatives that are based on the technologies described in the following sections.

#### 2.2.2. Identity-Based Encryption (IBE)

The *Identity-based Cryptography* (IBC) [53] was initially proposed as a certificateless public key cryptography alternative, in which a piece of public information associated with an entity (e.g., its email address) is used as the public key. Under the main foundations of IBC, [13] proposed an *Identity-based Encryption* (IBE) scheme, which allows to encrypt a message under a character string that is considered as the identity of the message's recipient. Consequently, unlike more traditional PKI-based approaches infrastructure, an entity does not need access to the recipient's certificate to encrypt amessage, simplifying key management tasks and reducing the overhead related to certificates transmission. Moreover, a central entity (*i.e.*, a *Trusted Third Party* (TTP)) is usually responsible for generating private keys and send them to the corresponding entities. This process of key generation requires an authentication mechanism, by which an entity demonstrates certain identity. Then, these keys can be used by entities for a secure communication without the need to involve the TTP. An IBE scheme usually consists of four main steps:

- Setup (*k* → {*params*, *MSK*}). This algorithm is executed only once by the TTP. It takes a security parameter *k* as input, and generates a master secret key *MSK*, as well as a set of public parameters *params* of the system.
- Extract ({*MSK*, *params*, *ID*} → *SK_ID_*). This phase is performed by the TTP when an entity requests a private key. It takes the master key *MSK*, the public parameter *params*, and a characters string *ID* as an input, generating a private key *SK* associated to the identity *ID*.
- Encrypt ({*params*, *M*, *ID*} → *CT*). This step is executed by an entity that wants to send an encrypted message *M* to an entity whose public identity is reflected by *ID*. The result of the algorithm is a ciphertext *CT*.
- Decrypt ({*params*, *CT*, *SK_ID_*} →*M*). Once the recipient gets *CT*, it tries to get *M* by using the public parameters *params* and its private key *SK_ID_*.

While IBE provides relevant features to be leveraged in different scenarios, in this work, it is used as one of the proposed alternatives, in order to enable an anonymous access control mechanism for IoT scenarios. Section 4.2 will provide a detailed description of this approach.

#### 2.2.3. Ciphertext-Policy Attribute based Encryption(CP-ABE)

The *Fuzzy Identity-based Encryption* (Fuzzy IBE) was introduced by [54] as a generalization of IBE, in which an identity is considered as a set of descriptive attributes. This scheme is considered as the first *Attribute-based Encryption* (ABE) scheme, and it is receiving attention from the research community due to its flexibility to enable encrypted communications among groups of entities. Based on ABE, two alternative approaches were proposed. In the *Key-Policy Attribute-Based Encryption* (KP-ABE) scheme [55], a message is encrypted under a set or list of attributes, while private keys of entities are associated with combinations or policies of attributes. In contrast, in the *Ciphertext-Policy Attribute-Based Encryption* (CP-ABE) scheme [14], a ciphertext is encrypted under a policy of attributes, while keys of participants are associated with sets of attributes. Thus, CP-ABE could be seen as a more intuitive way to apply the concepts of ABE; on the one hand, a data producer can exert greater control over how the information is disseminated to other entities. On the other hand, a user's identity is intuitively reflected by a certain private key. The main CP-ABE algorithms are described as:

- Setup (λ → {*PP*, *MSK*}). It takes an implicit security parameter λ as an input. The algorithm generates the public parameters *PP*, which are common to all users of the system (for example, the universe of attributes *U*) as well as a master secret key *MSK* which will be used by the TTP to generate secret keys for participants.
- KeyGen ({*MSK*, *A*} → *SK_A_*). After an entity proves it has a certain set of attributes *A*, the algorithm takes the master key *MSK* and the set *A* as an input. The result is a private key *SK_A_*.
- Encrypt ({*PP*, *M*, *PT*} → *CT*). It takes the message *M*, public parameters *PP* and a decryption policy *PT* representing subsets of attributes, which are allowed to decrypt *M*. The result of this algorithm is a ciphertext *CT* containing *PT*.
- Decrypt ({*PP*, *CT*, *SK_A_*} → *M*). The decryption algorithm takes as input the public parameters *PP*, the ciphertext *CT* with a *PT* associated, and a private key *SK_A_*. If the set *A* satisfies the policy *PT*, the subject will be able to decrypt *CT* with *SK_A_*.

In this work, CP-ABE is used as a different alternative to prove the possesion of a specific DCapBAC token in a privacy-preserving-way For this purpose, the original capability token form has been extended with a new field, and it will be explained in Section 4.1.

#### 2.2.4. Identity Mixer (Idemix)

Anonymous Credential Systems, such as Idemix [15] and U-Prove[56], enable selective disclosure of identity information to provide anonymity. Unlike traditional identity management mechanisms (e.g., based on X.509 certificates), users are able to obtain credentials to prove that they satisfy certain identity attributes, without disclosing any other additional information. Identity attributes are encoded into cryptographic proofs that can be selectively disclosed in a fine-grained way. Both U-Prove and Idemix are being explored in the scope of the ABC4Trust EU project, which is intended to provide a common architecture for privacy-ABC systems [57] In particular, Idemix is based on the *Camenisch-Lysyanskaya* (CL) signature scheme [58], which allows to prove the possession of a signature avoiding the disclosure of underlying messages, or even the signature itself, by using zero-knowledge proofs. Idemix operates with two main protocols:

- The **issuance protocol** is used by the *recipient* to obtain a credential from the *issuer*. During this process, the recipient selects a master secret key that will be encoded into every credential. The recipient computes a cryptographic message with the set of attributes, which are signed by the issuer and included in the credential.
- The **proving protocol** is initiated by the *prover* in order to prove the possession of a certain credential to the *verifier* entity, without revealing the credential itself. For this purpose, the prover creates a cryptographic proof by defining a proof specification (following a XML-based language), which is sent to the verifier. It contains a proof of the CL-signature possession, which ensures that the prover holds a credential that was signed by the issuer.

Furthermore, Idemix allows an entity to generate pseudonyms that demonstrate the possession of its master secret. During the proving protocol, the proof generated by the suject proves to the target the knowledge of the secret associated to a pseudonym, as well as the fact that such a pseudonym is based on the user's master secret key. That is: *nym* ← *g^m^*^_1_^ *h^r^* where *g* and *h* are public common group parameters, *m* is the master secret key and r is a randomization exponent. Then, the prover computes a challenge using a hash function, by considering the context and the computed proofs, *i.e.*, the CL proof and the pseudonym proof, as an input. Specificall, the prover computes random t-values of the form *t* ≔ *g^r^*, it get a challenge *c* ≔ *H*(…∥*t*), and finally it computes a response s-values of the form *s* ≔ *r* − *cα* to prove knowledge of *α*. Then, the target entity (verifier) computes the s-values that are hashed and compared against *c*. It should be noticed, that although it is not required in our proposal, Idemix also allows to prove more complex kind of proofs, which deal with inequalities and logic operators (AND, OR, NOT) over the attributes contained in a credential.

In this work, we use Idemix as one of the three proposed approaches to link a capability token to a pseudonym, in order to enable subjects to prove the possession of such token, while concealing their real identity. For further information about the Idemix cryptographic scheme, the reader is referred to the Idemix specification [15].

## 3. Security Framework for the IoT

**Figure 1:**
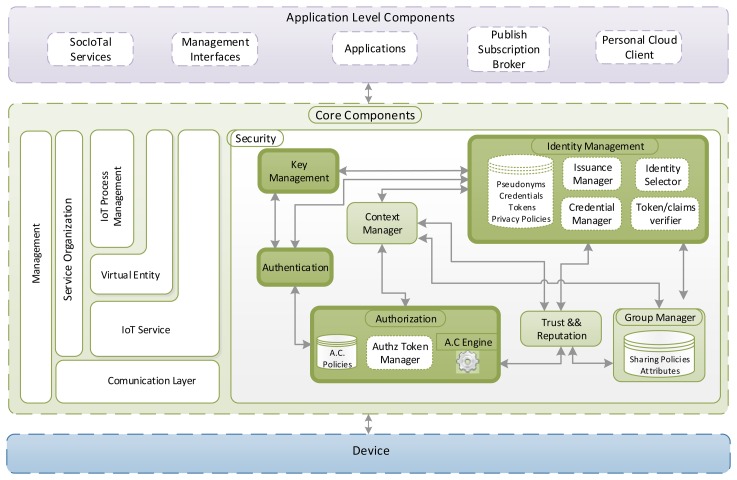
Architectural Reference Model (ARM)-based Security Framework for the Internet of Things (IoT).

The challenging complexity for a secure management of IoT smart objects imposes the need to consider architectural approaches, taking into account the inherent requirements of the application of security and privacy-preserving mechanisms on IoT scenarios. Indeed, the huge potential of IoT may be threatened if security and privacy concerns are not taken into account from the beginning, supporting aspects such as *privacy by design* [59], and *data minimization* [60] principles, in order to give people maximum control over their personal data. IoT-A [61] was a large-scale European project focused on the design of an *Architectural Reference Model* (ARM), in order to optimize the interoperability among isolated IoT domains to create a global ecosystem of services under a common view. This promoted additional initiatives adopting ARM as the starting point of design activities, favoring the alignment of architectures and enabling to reuse functionalities and components among different application domains. However, security and privacy concerns are not the main focus of such architectures. In contrast, our ARM-compliant security framework [18] addresses these requirements by instantiating and extending the security functional group of ARM, which promotes its applicability and interoperability in a wide range of IoT scenarios, in which security and privacy are required. Figure 1 shows our ARM-compliant framework, in which we highlight the main components that are addressed in this work.

**Figure 2:**
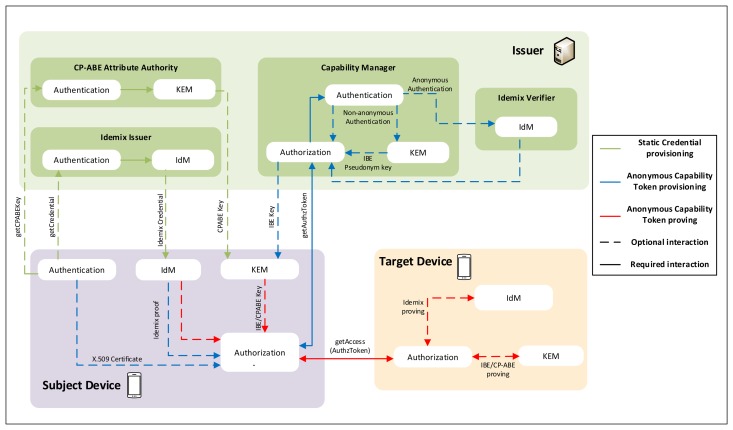
Architecture and framework interactions of the proposed approach.

Under the common view of our IoT security framework, Figure 2 shows the instantiation of the core functional components into hardware components, as well as the required interactions involved in our proposal. Specifically, three main actors are considered: *a target* entity hosting services and resources, a *subject* entity trying to get access to them, and an *issuer* entity, which is responsible for managing and delivering security credential in order to enable a secure and privacy-preserving communication between subject and target.

According to the figure, the required interactions are split into three main stages. During the first phase, the subject tries to get authentication credentials associated with its identity. In particular, depending on the alternative being used, it can obtain a CP-ABE key, through the *CP-ABE Attribute Authority*, or an Idemix credential, via the *Idemix Issuer*. In either case, the functionality of this phase requires an explicit authentication process, in which the subject proves it possesses a specific set of identity attributes. Then, during the second stage, the subject tries to acquire anonymous capability tokens from the issuer in order to get access to the target. This process requires authentication and authorization tasks through the corresponding functional components. While the authorization process is common to the three alternatives through the *Capability Manager* entity, the authentication can be carried out in a privacy-preserving way via the *Idemix Verifier*. Finally, during the third stage, the subject attempts to access a resource of the target entity. For this purpose, it makes use of the anonymous capability token, which was obtained in the previous process. However, the process to demonstrate the subject is the entity associated with that token depends on the alternative being used (*i.e.*, IBE, CP-ABE or Idemix). The next section will provide a detailed description of these alternatives.

## 4. Privacy-Preserving DCapBAC Alternatives

Under the main technologies described, as well as the instantiation of our security framework, in this section we provide a detailed description of the alternatives proposed in this paper to provide an anonymous access control mechanism for IoT scenarios.

### 4.1. Anonymous DCapBAC

As already described in Section 2, the access control mechanism proposed in this work is based on the DCapBAC model. Although DCapBAC has already been proven as an efficient and flexible access control approach for IoT environments, it does not support privacy-preserving features. Indeed, the entity that uses the DCapBAC token is unequivocally identified due to the use of the public key as identity. Therefore, prior to the description of the alternatives proposed in this work, the specification of an anonymous authorization credential is required.

The Listing 1 shows an example of the anonymous capability token (*Anonymous DCapBAC token*), which has been designed for this work. Unlike the original approach, in which the subject field (“su”) contained the value of the public key of the subject, in our proposal, this value is associated with two elements:

- **pseudonym** (“ps”) (mandatory). It is a 16 bytes alphanumeric string, which contains the value of the pseudonym associated with a specific capability token.
- **policy** (“po”) (optional). It is a character string specifying the combination of identity attributes that must be satisfied by the subject's identity. Specifically, the format of this field is:
  $$\text{attributetype}:\text{attributevalue}{\left(\left(\mathrm{AND}|\mathrm{OR}\right)\text{attributetype}:\text{attributevalue}\right)}^{+}$$

As we will discuss in the next sections, the *ps* field is used in the IBE and Idemix to demonstrate that a subject is an entity associated to a certain capability token, while its privacy is preserved. In the case of the CP-ABE alternative, the *po* field is used as a CP-ABE policy, which must be satisfied by the subject's identity when making an access request. Furthermore, the *ps* field is used in the three approaches for accountability reasons, in order to mitigate the misuse or abuse of the anonymity.


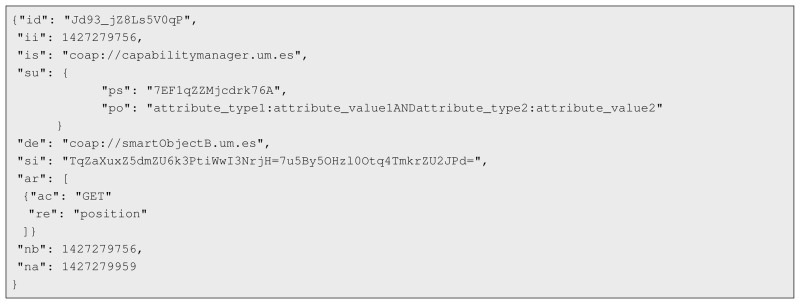


Listing 1: Anonymous DCapBAC Token example.

### 4.2. IBE-Based Anonymous DCapBAC

For the three alternatives, we considered smart objects have an X.509 certificate, which was given during the provisioning or manufacturing process of the smart object. In the IBE-based approach, two main phases are distinguished. During the first stage, the subject entity gets an Anonymous DCapBAC token, as well as an IBE key associated with the pseudonym value, which is contained within the *ps* field of such token. Both credentials are then used during the second stage to access the resource as the privacy of the applicant is preserved.

**Figure 3:**
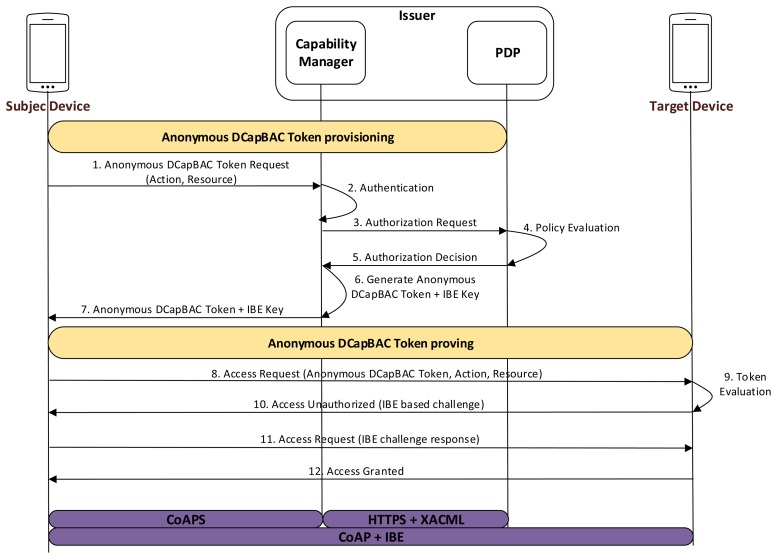
Identity-Based Encryption (IBE)-based Anonymous DCapBAC interactions.

Figure 3 shows the main interactions of the IBE-based Anonymous DCapBAC approach. Firstly, during the *provisioning* stage (1–7 messages), the Subject entity requests an Anonymous DCapBAC Token to the Capability Manager by using CoAP, and specifying the *action* and the target *resource* for which it requests such credential. However, before sending this request, a secure channel is established between both entities through DTLS with certificate-based mutual authentication. Specifically, we assume that the certificate of the Subject entity contains a set of identity attributes (e.g., manufacturer or device class), as part of the *subjectDirectoryAttributes* extension within an X.509 certificate [62]. Optionally, attribute certificates [63] could be employed together identity certificates for the token generation stage, including additional attributes for authorization purposes. These attributes, along with the action and resource specified in the CoAP request, are used by the Capability Manager to build a XACML request, which is sent to the PDP within the body of an HTTP request. Then, this request is evaluated against the set of XACML policies contained in the PDP and, as a result, an authorization decision is sent back. In the case of a *“Permit”* decision, the Capability Manager's task is twofold: first, it generates an Anonymous DCapBAC token *ADT* under the format described in the previous section. In particular, this credential uses the *ps* field with a randomly generated *pseudonym* value. Furthermore, the Capability Manager generates an IBE key associated to such pseudonym by using the *Extract* algorithm, which was described in Section 2.2.2 as follows:

$$\left\{\text{MSK},\text{params},\text{pseudonym}\right\}\to {SK}_{\text{pseudonym}}$$

Both *ADT* and *SK_pseudonym_* are sent to the Subject within the payload of a CoAP response with 2.05 Code (Content). Otherwise, in the case of a negative authorization decision from the PDP, the Capability Manager builds CoAP response with 4.01 Code (Unauthorized), which is sent to the Subject entity.

Then, during the proving stage, the Subject entity attempts to perform an action on a specific resource being hosted by the Target device. However, before making this request, it generates a symmetric key (e.g., AES) to protect the rest of messages required for the exchange, as well as to reduce the use of other expensive cryptographic operations. For this purpose, firstly, the subject generates a session key *SK_session_*, which is in turn encrypted with the public key of the target *PK_Target_*, and sends the following message (message 8):

$$8.\text{Subject}\to \text{Target}:\left[\text{EncryptPK}\left({PK}_{\text{Target}},{SK}_{\text{session}}\right),\text{EncryptSK}({SK}_{\text{session}},\left(t∥\text{action}∥\text{resource}∥\text{ADT}\right)\right]$$

where *t* is a timestamp, *action* is a CoAP method (*i.e.*, GET, POST, PUT, DELETE), *resource* is the URI resource of the target entity, and *ADT* is the anonymous token obtained in the previous stage. The result of these cryptographic operations is sent within the payload of the initial CoAP request.

Then, the target entity decrypts *SK_session_* with its private key *SK_Target_*, and uses *SK_session_* to decrypt the *action*, *resource* and *ADT* fields. These values are used by the Target to evaluate the capability token, as described in our previous work [9]. However, unlike DCapBAC proposal, in the case of a successful token evaluation, the Target challenges the subject entity in order to demonstrate it has an IBE key associated to the pseudonym value within the ps field of ADT. For this purpose, it uses the encrypt algorithm of Section 2.2.2, and sends the following message:

10.Target→Subject:[EncryptIBE((params,(M∥t),pseudonym)]

where *t* is a timestamp, *pseudonym* is the value contained in the *ps* field of *ADT*, and *M* ∈ *S* is a random element of the message space *S* = {0, 1}*^N^*. The resulting encrypted value is sent to the subject entity by using an additional CoAP header called *AUTHREQ*, within a 4.01 (Unauthorized) response.

After receiving this message, the subject entity tries to build a response to the challenge sent by the target. For this purpose, he runs the algorithm Decrypt described in Section 2.2.2 by using the IBE key *SK_pseudonym_* obtained during the provisioning stage. The result of the algorithm is included in a new header called *AUTHRES*, and sent to the target within a CoAP request:

$$11.\text{Subject}\to \text{Target}:\left[\text{EncryptSK}\left({SK}_{\text{session}},\left(\text{DecryptIBE}\left(\text{params},\left(M∥t\right),{SK}_{\text{pseudonym}}\right)∥t\prime \right)\right)\right]$$

Finally, the target checks that the challenge response from the subject matches M. In the case of a correct value, the target sends the requested resource to the subject in a CoAP response with 2.XX code (Success):

$$12.\text{Target}\to \text{Subject}:\left[\text{EncryptSK}\left({SK}_{\text{session}},\text{resourcevalue}∥t\right)\right]$$

### 4.3. CP-ABE Based Anonymous DCapBAC

**Figure 4:**
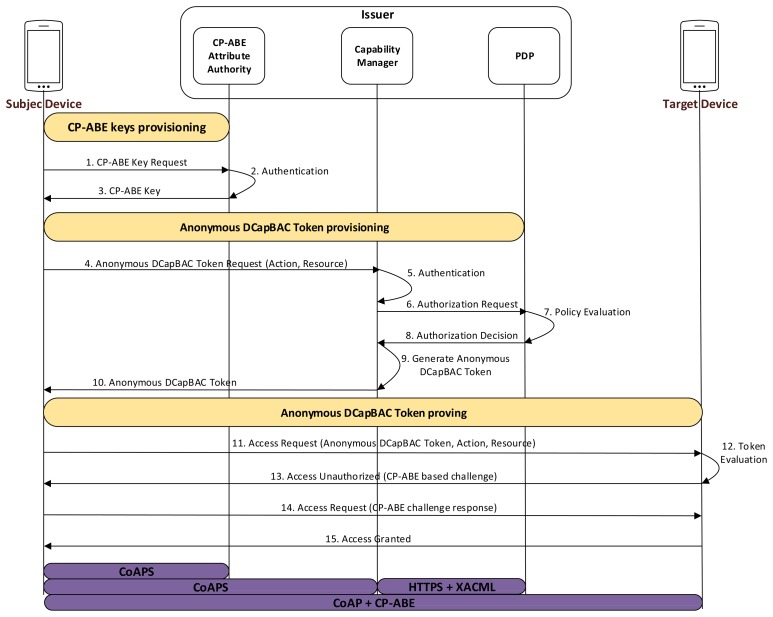
Ciphertext-Policy Attribute-Based Encryption (CP-ABE) based Anonymous DCapBAC interactions.

In this alternative, an Anonymous DCapBAC token, along with a CP-ABE key are used to demonstrate the possession of a certain authorization credential, while privacy of the requester smart object is still preserved. As shown in Figure 4, three main stages are differentiated for this approach. Firstly, a smart object, acting as a subject entity, obtains a CP-ABE key *SK_A_* associated to a set of identity attributes *A* (message 1–3). This phase requires an explicit authentication process by which the subject device proves that it has a certain set of attributes (e.g., those attributes contained in its X.509 certificate). In this case, we propose to use CoAP-DTLS exchange in order to deliver the CP-ABE key over a secure channel. However, unlike the previous approach, in which a new IBE key is generated for each token, it should be pointed out that this process is required only once (or in the case the CP-ABE key has expired or has been revoked).

Then, as in the previous approach, the subject entity obtains an Anonymous DCapBAC token to access a target device (messages 4–10). However, unlike the IBE-based alternative, the anonymous capability token contains a combination of identity attributes (or partial identity) within the po field, which must be satisfied by the subject entity when trying to access the target device. This combination of attributes, although it is imposed by the issuer entity, could be dependent on information (e.g., contextual conditions), which is sent by the Target. Moreover, although the ps field is still used to facilitate the traceability and accountability of the anonymity, in this case, the generation of new cryptographic material is not required, since the CP-ABE key (previously obtained) will be used as identity credential during the Anonymous DCapBAC token proving stage.

This phase is based on the process that was described in the previous section. Therefore, the subject entity generates a session key SKsession, which is in turn encrypted with the public key of the target, and sends:

$$11.\text{Subject}\to \text{Target}:\left[\text{EncryptPK}\left({PK}_{\text{Target}},{SK}_{\text{session}}\right),\text{EncryptSK}({SK}_{\text{session}},\left(t∥\text{action}∥\text{resource}∥\text{ADT}\right)\right]$$

Once the target entity has evaluated the capability token, it extracts the value of the *po* field, and challenges the subject entity to demonstrate that it has an identity (*i.e.*, a CP-ABE key), satisfying the identity attributes combination *cpabepolicy*, which is contained in such field. For this purpose, it makes use of the *Encrypt* algorithm of Section 2.2.3, and sends:

13.Target→Subject:[EncryptCPABE(PP,(M∥t),cpabepolicy)]

where *t* is a timestamp, *cpabepolicy* is the value contained in the *po* field of *ADT*, and *M*∈*G_T_* is a random group element from a pairing *e* : *G*_1_×*G*_2_→*G_T_*. The resulting encrypted value is sent to the subject entity by using an additional CoAP header called *AUTHREQ*, within a 4.01 (Unauthorized) response.

Upon receiving this message, the subject entity tries to build a response to the challenge sent by the target. For this purpose, he runs the algorithm *Decrypt* described in Section 2.2.3 by using the CP-ABE key *SK_A_* obtained during the initial stage. As in the IBE-based alternative, the result of the algorithm is included in a new header called *AUTHRES*, and sent to the target within a CoAP request:

$$14.\text{Subject}\to \text{Target}:\left[\text{EncryptSK}\left({SK}_{\text{session}},\left(\text{DecryptCPABE}\left(PP,\left(M∥t\right),{SK}_{A}\right)∥t\prime \right)\right)\right]$$

The target entity checks that the challenge response from the subject matches M. If so, it sends the requested resource to the subject in a CoAP response with 2.XX code (Success):

$$15.\text{Target}\to \text{Subject}:\left[\text{EncryptSK}\left({SK}_{\text{session}},\text{resourcevalue}∥t\right)\right]$$

### 4.4. Idemix-Based Anonymous DCapBAC

**Figure 5:**
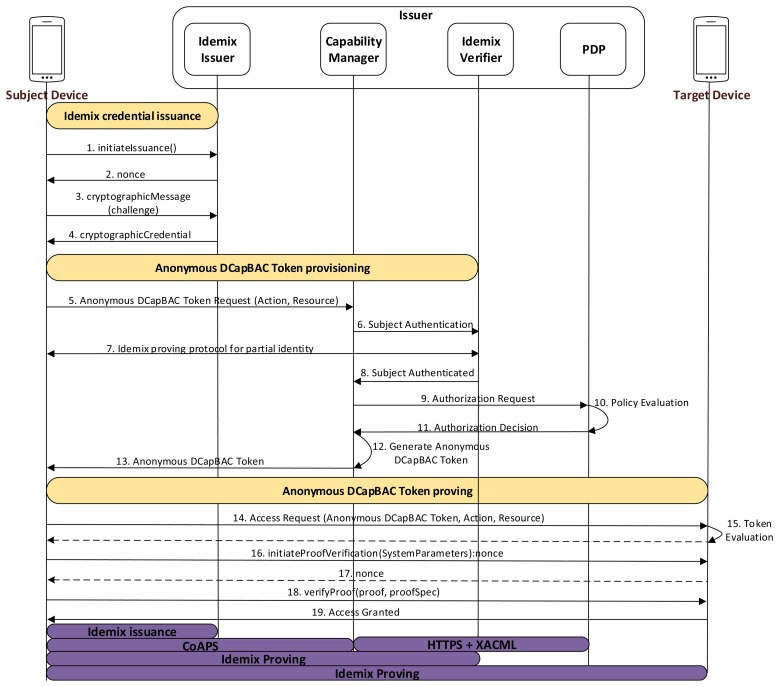
Idemix-based Anonymous DCapBAC interactions.

This approach allows the subject device to use its Idemix credential to obtain an Anonymous DCapBAC token from the Capability Manager in a privacy-preserving-way. Furthermore, Idemix is also used to enable the subject to use the Idemix proof protocol, in order to demonstrate that it is the entity linked to the pseudonym included in the capability token. Figure 5 shows the main stages that have been identified for this alternative.

In the first stage, the subject device obtains an Idemix credential from the Idemix Issuer, as part of the Issuer entity. The Idemix credential will be used in the second stage to derive a proof of possession of the attributes that are hold by the subject in the credential. During this process, the subject and issuer entities carry out the *Idemix Issuance Protocol* (messages 1–4). For this purpose, they must agree on a common group of parameters, system parameters, as well as the credential specification structure. These configuration properties are put in common by using public files, which are accessible via well-known URIs. In addition, the first time the subject is authenticated against the Issuer (that is, the subject does not have an Idemix credential yet), the former needs to demonstrate latter who it claims to be. To this aim, we propose to use traditional certificate-based authentication through CoAP-DTLS.

Once the subject device obtains an Idemix credential, it can be used to get capability tokens in a privacy-preserving way. For this purpose, it contacts with the Capability Manager entity indicating the specific resource and action to be performed on the target device. Then, the Capability Manager contacts with the Idemix Verifier entity, who is responsible to authenticate the subject through the Idemix Proving Protocol (interaction 7). During this message exchange, the subject generates a cryptographic proof *cpa* in order to demonstrate a specific combination of identity attributes, as well as a cryptographic proof *cps* of a pseudonym, which is randomly generated by the subject.

After completion of the proving protocol, the Idemix Verifier sends both proofs to the Capability Manager for authorization purposes (message 8). Then, it extracts the identity attributes of the *cpa* proof, and builds a XACML request with such attributes, as well as the action and resource obtained in the message 5. As in the previous alternatives, in the case of a “*Permit*” decision from the PDP, the Capability Manager generates an Anonymous DCapBAC token. However, in this case, the value of the *cps* proof is used as the *ps* field value.

During the Anonymous DCapBAC token proving stage, the subject device uses the capability token previously obtained to get access to a resource of a target device. For this purpose, as in the previous alternatives (message 11 of Section 4.3), it generates an initial CoAP request, in which it includes:

$$14.\text{Subject}\to \text{Target}:\left[\text{EncryptPK}\left({PK}_{\text{Target}},{SK}_{\text{session}}\right),\text{EncryptSK}({SK}_{\text{session}},\left(t∥\text{action}∥\text{resource}∥\text{ADT}\right)\right]$$

Upon a successful token evaluation, the target entity extracts the pseudonym value contained in the ps field, and requests the subject to prove that it is the entity associated with such token. To this aim, both entities accomplish the Idemix Proving Protocol, which allows the subject to prove it is the entity associated to the capability token, and it has a valid credential issued by the Issuer, while concealing any other identity attributes. Firstly, the subject device initiates the proving proof protocol specifying the common system parameters to be used by both parties (message 16). The target device responses with a nonce, which is used by the subject device to generate the proof. Then, the proof is built by the subject, which loads his Idemix credential (previously obtained during 1–4 steps), and generates the proof according to the proof specification format. This proof contains a pseudonym subproof along with the CL signature subproof. (It should be pointed out that Idemix allows that the attribute values can be unrevealed to the target device). Then, the subject makes use of the *verifyproof* interface by sending the proof specification, as well as the generated proof (message 17). Then, the target device verifies the proof including the pseudonym subproof and the CL subproofs. Then, if the pseudonym is valid, the access is granted, and consequently, a CoAP response with 2.XX code (Success) is sent to the subject device:

$$19.\text{Target}\to \text{Subject}:\left[\text{EncryptSK}\left({SK}_{\text{session}},\text{resourcevalue}∥t\right)\right]$$

## 5. Evaluation Results

According to the different anonymous access control alternatives previously described, in this section we analyze and compare the performance of these approaches. We focus the evaluation on the Anonymous DCapBAC Token proving stage, in which a subject device must demonstrate that is the entity associated to such token in a privacy-preserving way. For token evaluation results, the reader is referred to [9,52], in which a similar testbed is employed. Both entities were implemented as v.4.1.2 Android applications on a Smartphone LG-P760 with a dual-core 1GHz Cortex-A9 processor and 1GB RAM. Below, we show the results of each of the three alternatives separately.

### 5.1. Performance of the IBE-Based Anonymous DCapBAC

**Figure 6:**
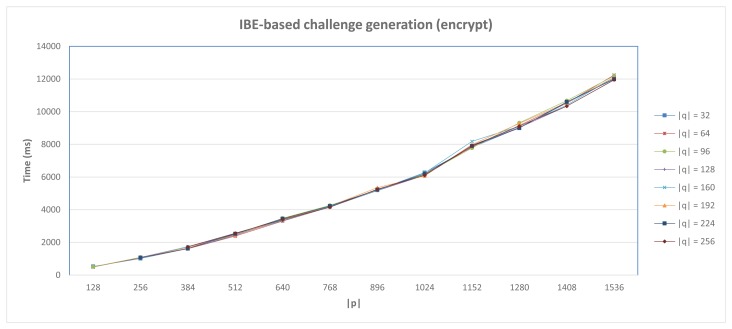
IBE-based Anonymous DCapBAC. Challenge generation performance.

For the IBE-based approach, performance results are primarily influenced by the desired security level, which depends on cryptographic parameters being employed. Specifically, we have made use of the jpair library [19], which has been deployed on the testbed previously described. This library provides an implementation of the Boneh-Franklin (BF) IBE scheme [13] by using type A pairings, which are built on the supersingular curve *y*^2^ = *x*^3^ + *x* over the field *F_p_* for some prime *p* = 3 *mod* 4. In this case, let *p* be the prime order of *F_p_*, and *E*(*F_p_*), the additive group of points of affine coordinates (*x*, *y*) with *x*, *y* in *F_p_*, that satisfy the curve equation, *q* represents the order of the cyclic subgroup of interest in *E*(*F_p_*). Under these assumptions, we used distinct configurations in order to evaluate the performance of this alternative for different security levels. At this point, it should be pointed out that the security level of this stage also depends on other cryptographic material, which is used during the message exchange described in Section 4.2. Consequently, in the case of symmetric and public key cryptographic operations, we used keys according to the IBE parameters, in order to maintain the same security level during the whole process. As stated by [64], the security level of the BF scheme depends on the size of primes *p* and *q*. Thus, Figure 6 shows the average delay for the challenge generation step (fluctuating *p* and *q* size), corresponding to the delay required to generate the message 10 (*EncryptIBE* operation), described in Section 4.2. According to the figure, the delay for this task mainly depends on the bits number of *p* (denoted as |*p*|).

**Figure 7:**
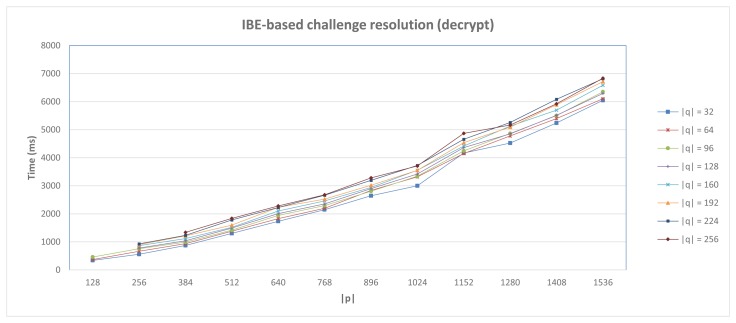
IBE-based Anonymous DCapBAC. Challenge resolution performance.

Furthermore, Figure 7 shows the delay required in the case of the decryption operation, which is used to build the message 11 defined in Section 4.2 (*DecryptIBE* operation).

These values were obtained by averaging the results of 10 executions for such operations. According to it, the IBE-based alternative provides reasonable times for low security levels, but impractical in the case of higher levels, especially in scenarios where direct interaction between devices is required, as proposed in this work. In particular, considering a 80-bits security level (|*p*| = 512, |*q*| = 160), the minimum security level recommended by NIST [65], the delay required for the encryption operation is 2525 ms, whereas for decryption, it takes 1516 ms. In the case of a 128-bits security level (|*p*| = 1536, |*q*| = 256), it requires 11,951 ms and 6833 ms, respectively. Therefore, in the case of scenarios with high security level requirements, the application of cryptographic optimizations, (e.g., by using pre-computations [66]) could be a valuable contribution in order to design IBE-based security mechanisms for constrained devices.

### 5.2. Performance of the CP-ABE Based Anonymous DCapBAC

The evaluation of the CP-ABE based approach is focused on the process that is required to demonstrate the possession of an Anonymous DCapBAC token by using CP-ABE. The performance of this scheme, in addition to the desired security level, also depends on the number of attributes used to define the access policy (contained in the *po* field of the token). We deployed a Java-based CP-ABE library on Android, which we used as proof-of-concept of the proposed mechanism in Section 4.3. This library [67] implements the CP-ABE scheme provided by [14], which is built over the *Java Pairing Based Cryptography* library (jPBC) [68]. As in the IBE-based alternative, this library is based on the use of type A pairings, consequently, the security considerations defined in the previous alternative are also applicable to this evaluation [69].

**Figure 8:**
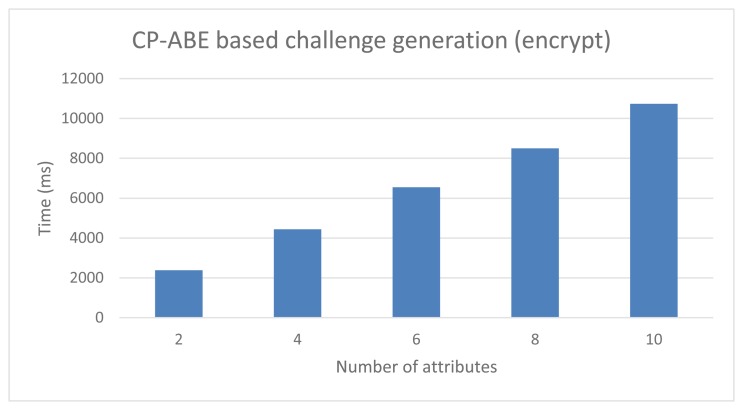
CP-ABE based Anonymous DCapBAC. Challenge generation performance.

**Figure 9:**
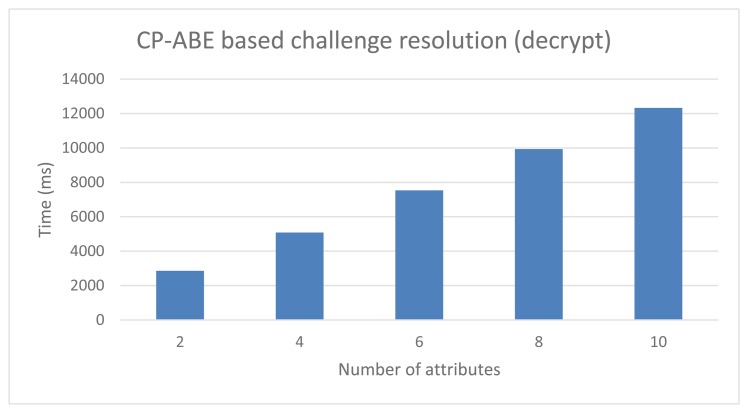
CP-ABE based Anonymous DCapBAC. Challenge resolution performance.

Figure 8 shows the performance results for the challenge generation process (step *EncryptCPABE* of message 13), while the values of the resolution process are shown in Figure 9 (step *DecryptCPABE* of message 14). These results were obtained by modifying the number of attributes defined in the access policy from 1–10, since we consider this range expressive enough for most scenarios and use cases. Furthermore, at this point, it should be pointed out two aspects of this evaluation. On the one hand, these values were achieved by considering an 80-bits security level (*i.e.*, |*p*| = 512 and |*q*| = 160), which is suitable for scenarios with medium security requirements. On the other hand, the policies defined for this evaluation only consider AND connectives for one level of attributes. Therefore, the CP-ABE key used by the subject device to resolve the challenge, should be associated to (at least) all the attributes that were used to define the access policy in the encryption process.

According to the results, the time required for each cryptographic algorithm depends directly on the number of attributes being used. Thus, under these considerations, in the case of a 2-attributes access policy, the encryption operation takes 2381 ms, while the decryption process requires 2858 ms. However, in the case of access policies defined on a larger set of attributes, the performance is considerably worse. For example, with a 10-attributes policy, the delay required for encryption and decryption operations is 10,734 and 12,333 ms, respectively. This is mainly due to the computational complexity of pairings cryptographic operations, as well as the use of a tree data structure, which is used to represent the access policy in the CP-ABE library used.

While the scheme can be still used in scenarios without direct interaction between devices, these results obtained with this specific library make it unfeasible the application of CP-ABE on the proposed mechanism, in the case of access policies defined on a broad set of attributes. Therefore, the implementation of a lightweight CP-ABE scheme through the application of cryptographic optimizations over pairings, as well as the use of simpler data structures to represent access policies, could serve as basis for the design of a CP-ABE scheme suitable for constrained devices.

### 5.3. Performance of the Idemix-Based Anonymous DCapBAC

The testbed consists on evaluating the performance of the Idemix proving protocol, which is carried out by the subject device to proof that it is in possession of a valid credential and pseudonym, while anonymity is preserved. In the testbed, the Idemix proof that is sent to the target device, contains the pseudonym (the same included in the capability token), as well as an incremental amount of unrevealed attributes (different amount in different tests). The implementation relies on the Idemix Java library (version 2.3.0) ported to Android by [70]. It is worth mentioning that the testbed uses Android SDK 1.7 and the RSA secret key length is established to 1024 bits (*i.e.*, 80-bits security level), in order to compare the results with previous alternatives.

**Figure 10:**
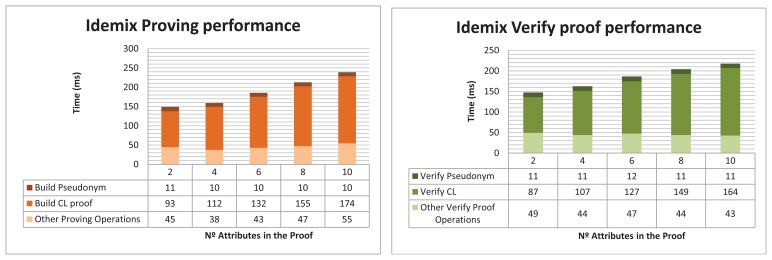
Idemix-based Anonymous DCapBAC. Privacy preserving access performance.

Figure 10 shows two graphs that sum up the performance times obtained in the Idemix testbed. The left chart shows the time required by the subject to build the proof. The total build proof operations made in the subject side are split in 3 different series, in order to be able to show the times required to build the pseudonym subproof and the CL subproof, which are the most heavy tasks in the whole proving protocol. The series *Other proving operations* encompasses, among others operations, the time require to load the credential previously obtained, initiate the verification process to obtain the nonce, parse and validate the proof specification, as well as generate the challenge. The X-axis in both charts represent the amount of attributes used in the proof. As can be seen in the left graph, the time required to build the pseudonym subproof is barely the same regardless of the amount of attributes in the proof. The CL subproof generation requires more computation time, since the proof contains a higher amount of attributes. The time required to perform other operations are usually steady across the different tests. Notice that the results does not include the network delay.

On the other hand, the Verify Proof operation, which is carried out by the target device, is shown in the right chart of the Figure 10. The total time required during the proof verify task is split again to show independently the time required to verify the pseudonym and the CL signature. As it was predictable, the times increases as the amount of attributes in the proof is higher. As can be seen, according to both charts, the verification operation against the build proof operation requires a slightly less computation time.

## 6. Discussion

The application of security and privacy-preserving mechanisms on IoT scenarios has to face significant challenges because of the need to manage millions of heterogeneous devices with the ability to interact each other. In this work, we have presented the design and evaluation of different cryptographic schemes in order to enable an anonymous and traceable access control mechanism for M2M-enabled IoT scenarios.

While the previous section provided a set of experimental results, in this section we compare and analyze the proposed alternatives, by considering such evaluation results, as well as other qualitative aspects related to privacy features (e.g., unlinkability and accountability) and practical aspects. Besides the original DCapBAC approach, we include two additional mechanisms in this comparison. On the one hand, *Traceable Anonymous Certificates* (TACs) [71] provide a mechanism for a privacy-preserving identity management by using pseudonyms that are included in an X.509 certificate. On the other hand, the *On-line Credentials* category [72] consists of well-known technologies and protocols, such as the *Security Assertion Markup Language* (SAML) [20] or OpenId [21], which are usually deployed on common scenarios of the current Internet.

Table 1 shows the analysis of these alternatives regarding privacy features and practicality aspects, which must be considered for a real deployment on IoT environments. The *Credential/Key issuance periodicity* aspect is related to the management of cryptographic material that is used to demonstrate the possession of an Anonymous DCapBAC token in a privacy-preserving way. However, it should be pointed out that the tokens themselves could be also generated according to a different issuance periodicity. While DCapBAC tokens have a lifetime that is indicated in the token itself, they can revoked by the Issuer entity due to different reasons (e.g., misuse of it). In this case, the smart objects that are affected by this process, that is, those devices that could receive such token to be validated, need to be aware of the revocation of such token. For this purpose, the well-known publish/subscribe pattern could be employed as an alternative to avoid continuous checkups of revoked credentials for each transaction. In this way, smart objects could be subscribed to a *revocation* service on the Issuer in order to receive notifications for revoked DCapBAC tokens. However, although it can reduce significantly communications overhead, a persistent communication between smart objects and Issuer is still assumed. Therefore, the analysis of more flexible and scalable alternatives for management and revocation aspects will be explored in future works. Regarding the proposed mechanisms, one CP-ABE key (or Idemix credential) can be used to demonstrate the possession of different Anonymous DCapBAC tokens. On the contrary, the IBE-based approach requires the generation of a new key for each capability token, which makes key management cumbersome for subject entities. In the case of On-Line credentials and TACs, this feature is variable depending on the anonymity level to be obtained. It should be noticed, that anonymity is considered as the property by which a subject is not unequivocally identifiable. Such property can be applied for the main both stages of the proposed alternative: the token provisioning and the proving stages. For example, if a subject only has one TAC (with a pseudonym associated), all the capability tokens for this entity should contain the same pseudonym, which would make subject's transactions linkable. In the case of the issuer generates a new TAC for each capability, this approach would be similar to the IBE-based alternative with the already mentioned drawbacks for key management. Moreover, the *Support for common security protocols* feature refers to the ability of the different approaches to use standard mechanisms for securing communications. In this case, unlike the alternatives presented in this work, DCapBAC, On-Line credentials and TACs approaches can be deployed over standard security protocols (e.g., TLS/DTLS) to obtain basic security features, since they are based on the use of X.509 certificates.

The next features are related to different privacy aspects of the approaches being considered. According to these parameters, On-Line Credentials provide a reasonable level of privacy. Indeed, SAML or OpenID technologies are currently used in different scenarios of the Internet, enabling advanced features such as *Single Sign-On* [73] or *identity federation* [74]. However, a common aspect of these solutions is that they require the involvement of a *Trusted Third Party* (TTP) during the capability token proving process. Therefore, in these approaches, it is supposed the target device has a direct and persistent communication with the issuer entity. While it has been accepted for scenarios of the current Internet, this assumption does not hold in IoT scenarios where interactions are carried out through *Low power and Lossy Networks* (LLNs), which prevents the design and development of M2M-enabled security solutions. Consequently, the issuer entity becomes a bottleneck, making subject's transactions linkable for that entity. Regarding the rest of alternatives, the Idemix-based approach provide the highest level of anonymity, since it allows to carry out the *Anonymous DCapBAC token provisioning* stage in a privacy-preserving way. Indeed, as explained in Section 4.4, a subject entity could use a cryptographic proof from an Idemix credential in order to obtain a capability token, while its privacy is preserved. Regarding the unlinkability feature, we consider the definition of that is provided by [75]. For the proposed alternatives, it means the target device could not sufficiently distinguish whether subject interactions are related or not. In addition to the alternative to be considered, it should be noted that the unlinkability level also depends on the granularity that is considered in the definition of access privileges to be included within a capability token. For example, if a token is associated to the pair (*resource, action*), all the transactions for the same pair would be linkable. Consequently, in scenarios with strong unlinkability requirements, one-time capability tokens could be considered. Moreover, the accountability of the anonymity is always preserved through the use of an issuer entity, which is responsible for generating capability tokens. Therefore, in the case of a target entity detects abuse of misuse of the anonymity, it can contact the issuer entity for the corresponding anonymity revocation tasks. At this point, it should be noticed that the functionality related to revocation tasks (credentials or anonymity) could be carried out by a different entity. In this case, such component and the Issuer should keep a strong trust relationship.

Furthermore, we have included practical aspects of the different alternatives, in order to assess their feasibility for a real deployment on IoT scenarios. In particular, the *DCapBAC token size* remains constant for all alternatives except in the case of the CP-ABE based approach, since the definition of a CP-ABE policy (*po* field in the token) depends on the number of attributes that are used to define the access policy. As shown in Section 4, the number of *Subject-Target interactions* remains low (4–6 messages) for the proposed alternatives. In the case of On-Line Credentials and TACs, this value depends on the specific protocol being used. Finally, the Delay aspect is related to the evaluation results that were obtained. As shown in the previous section, the IBE and CP-ABE based alternatives provide reasonable results for low security levels and a small set of attributes, respectively. However, in the case of scenarios with strong security requirements, the application of different cryptographic optimizations needs to be considered. Moreover, in addition to offer the highest level of privacy, the Idemix-based approach provided suitable results to be used even with resource-constrained devices. Therefore, this alternative is postulated as a feasible and promising methodology to enable an anonymous access control approach for a M2M-enabled IoT.

## 7. Conclusions

The realization of a secure and privacy-preserving M2M-enabled IoT must face serious technical and social challenges. Several aspects related to scalability, dynamism, heterogeneity or usability need to be addressed in the design of new, valuable and secure services to be exploited by society. Under the main foundations of certificateless public key cryptography and anonymous credential systems, we have presented the design of three different mechanisms to enable an anonymous and traceable access control approach. Furthermore, these mechanisms have been developed and evaluated in order to assess their suitability for a real deployment on constrained device. According to the presented results and the security discussion, the proposed Idemix-based access control alternative provides advanced privacy features, as well as reasonable performance results, making it a promising approach to be considered on real scenarios. Moreover, its high level of flexibility and scalability, future fork is focused on the design and evaluation of secure and privacy-preserving group communication mechanisms based on certificateless public key cryptography.

## Figures and Tables

**Table 1:** Alternatives comparison.

	**DCapBAC** [9]	**On-Line Credentials** [72]	**TACs** [71]	**IBE Alternative**	**CP-ABE Alternative**	**Idemix Alternative**
**Credential/Key issuance periodicity**	Once/Until revoked	Once/One per token	Only once/One per token	One per token	Once/Until revoked	Once/Until revoked
**Support for common security protocols**	Yes	Yes	Yes	No	No	No
**Anonymous DCapBAC token proving**	No	Yes	Yes	Yes	Yes	Yes
**Anonymous DCapBAC token provisioning**	No	Yes	No	No	No	Yes
**Non TTP involvement during DCapBAC token proving**	Yes	No	Yes	Yes	Yes	Yes
**Unlinkability**	No	Variable	Variable	Yes	Yes	Yes
**Accountability**	Not applicable	Yes	Yes	Yes	Yes	Yes
**DCapBAC Token size**	Constant	Constant	Constant	Constant	Variable	Constant
**Subject-Target Interactions**	4	Variable	Variable	4	4	6
**Delay**	Very Low	Medium	Very Low	High	Very High	Low
